# Tripolar mitosis and partitioning of the genome arrests human preimplantation development *in vitro*

**DOI:** 10.1038/s41598-017-09693-1

**Published:** 2017-08-29

**Authors:** Christian S. Ottolini, John Kitchen, Leoni Xanthopoulou, Tony Gordon, Michael C. Summers, Alan H. Handyside

**Affiliations:** 1 The Bridge Centre, One St Thomas Street, London, SE1 9RY UK; 2 School of Biosciences, University of Kent, Canterbury, CT2 7NJ UK; 3 London Women’s Clinic, 113-115 Harley Street, Marylebone, London W1G 6AP UK; 4 Genesis Genetics, 705 South Main Street, Plymouth, MI 48170 USA; 5 Genesis Genetics, London Bioscience and Innovation Centre, 2 Royal College Street, London, NW1 0NH UK

## Abstract

Following *in vitro* fertilisation (IVF), only about half of normally fertilised human embryos develop beyond cleavage and morula stages to form a blastocyst *in vitro*. Although many human embryos are aneuploid and genomically imbalanced, often as a result of meiotic errors inherited in the oocyte, these aneuploidies persist at the blastocyst stage and the reasons for the high incidence of developmental arrest remain unknown. Here we use genome-wide SNP genotyping and meiomapping of both polar bodies to identify maternal meiotic errors and karyomapping to fingerprint the parental chromosomes in single cells from disaggregated arrested embryos and excluded cells from blastocysts. Combined with time lapse imaging of development in culture, we demonstrate that tripolar mitoses in early cleavage cause chromosome dispersal to clones of cells with identical or closely related sub-diploid chromosome profiles resulting in intercellular partitioning of the genome. We hypothesise that following zygotic genome activation (ZGA), the combination of genomic imbalance and partial genome loss disrupts the normal pattern of embryonic gene expression blocking development at the morula-blastocyst transition. Failure to coordinate the cell cycle in early cleavage and regulate centrosome duplication is therefore a major cause of human preimplantation developmental arrest *in vitro*.

## Introduction

Following *in vitro* fertilisation (IVF), normally developing fertilised human oocytes cultured *in vitro*, undergo a series of mitotic cleavage divisions, which subdivide the oocyte cytoplasm into proportionately smaller cells. Beginning with the fourth cleavage division between the 8- and 16-cell stages, four days following insemination (Day 4), the outer cells of the embryo compact, changing their shape to flatten out over the surface and maximise intercellular contact to form a morula. Finally, between Days 5 and 7, the embryo develops to the blastocyst stage in which the outer cells have now formed an epithelium of trophectoderm cells, specialised for implantation, which accumulates fluid in the blastocoel cavity, enclosing the inner cell mass from which the fetus is derived^1^. In contrast to the mouse, for example, in which most embryos develop successfully to the blastocyst stage in appropriate culture media, only about half of human embryos develop to the blastocyst stage, while the others arrest at cleavage stages and this is consistent across a range of media^2^. Various morphological abnormalities are associated with developmental arrest including cytoplasmic fragmentation, multinucleation, and unequal cleavage but the underlying causes remain poorly understood.

At the chromosomal level, many human embryos are aneuploid, mainly as a result of maternal age-related meiotic errors inherited from the oocyte^3^. Other aneuploidies arise following fertilisation in the early cleavage divisions by mitotic errors including anaphase lag and to a lesser extent non-disjunction resulting in chromosome mosaicism^4, 5^. Structural abnormalities, including partial gain and loss of chromosomes, are also common^6, 7^. Genomic imbalance of meiotic and mitotic origin is a major cause of pregnancy loss and is only rarely compatible with development to live birth^8^. Aneuploidy *per se*, however, does not appear to cause developmental arrest at preimplantation stages, although embryos with multiple aneuploidies of mitotic origin are less frequent at the blastocyst stage^9, 10^. Thus, in a large series of trophectoderm samples biopsied from blastocysts for aneuploidy screening, the incidence of aneuploidy ranged from about 30% to over 85% in women between the ages of 26 and 44 years^11^.

Another cause of aneuploidies of mitotic origin in early cleavage is likely to be abnormalities of mitosis itself. Multipolar, tripolar and monopolar spindles, have been identified by confocal laser scanning microscopy throughout cleavage and in the trophectoderm at the blastocyst stage^12^. Furthermore, time lapse imaging of developing embryos has confirmed that tripolar mitoses, division of a single blastomere directly into three daughter cells, is relatively common in the first three cleavage divisions^13, 14^. Assuming random recruitment of chromosomes to each axis of a tripolar spindle and normal bipolar segregation of sister chromatids, tripolar mitosis would theoretically reduce chromosome number from 46 to about 30 with a combination of normal biparental disomies, paternal and maternal monosomies and nullisomies in the daughter cells^15^.

Here we describe the use of genome-wide SNP genotyping and meiomapping of both polar bodies of oocytes to identify maternal meiotic errors and karyomapping to fingerprint all of the parental chromosomes (with the exception of the Y chromosome) in single cells from disaggregated arrested embryos and excluded cells from blastocysts^16–18^. Combined with retrospective time lapse imaging of development in culture, we demonstrate that tripolar and other abnormal mitoses in early cleavage result in clones of cells with identical or closely related sub-diploid chromosome profiles as predicted, effectively partitioning the genome between cell lineages.

## Results

Three couples undergoing IVF and genetic testing of embryos consented to both first and second polar bodies (PB1 and PB2, respectively) and trophectoderm biopsy in those embryos developing to the blastocyst stage. They also consented to follow up analysis of affected, chromosomally abnormal or arrested embryos that would not be used for clinical embryo transfer. Two couples (patients 1 and 2) had preimplantation genetic diagnosis (PGD) for a familial single gene defect combined with testing for chromosome aneuploidy and one couple (patient 3) had aneuploidy testing alone. In four IVF cycles (patient 1 had two IVF cycles), 61 mature oocytes arrested in metaphase of the second meiotic division (MII) underwent PB1 biopsy and intracytoplasmic sperm injection (ICSI) and 51 (84%) fertilised normally forming two pronuclei the following day (Day 1) (Table 1). Following biopsy of the PB2, embryos were cultured in an incubator fitted with time lapse imaging for up to 6 days (Day 7 post ICSI).

**Table 1:** Development of normally fertilised embryos, with two pronuclei, to Day 7 post intracytoplasmic sperm injection (ICSI).

Patient	Maternal age	Maternal genotype (rs2305957)	Cycle no	Fertilisation				Maternal meiotic errors	Development (2PN) post ICSI					
				MII	0PN	3PN	2PN		Arrest		Bls Day 5	Bls Day 6	Bls Day 7	Total Bls (%)
									Early	Late				
1	27	AA	1	12	1	0	11	7 (27)	0	5	4	1	1	7 (64)
			2	17	1	1	15		0	8	4	3	0	7 (47)
2	31	AA	1	13	1	1	11	1 (9)	0	5	6	0	0	6 (54)
3	43	AG	1	19	0	5	14	13 (93)	1	6	5	2	0	7 (50)
**Total**			**5**	**61**	**3**	**7**	**51**	**21 (41)**	**1**	**24**	**19**	**6**	**1**	**26 (51)**

PN: pronuclei, MII: Oocyte arrested at metaphase of the second meiotic division, ICSI: Intracytoplasmic sperm injection, Bls: Blastocyst.

Twenty six embryos (51%) developed to the blastocyst stage between Days 5–7 and approximately 3–5 trophectoderm cells were biopsied for genetic analysis. In addition, one or more cells which had been excluded from seven blastocysts and were loosely attached to the outer surface, were removed and further analysed. The biopsied blastocysts were then cryopreserved by vitrification for later clinical use. Of the remaining 25 embryos, one arrested on Day 2 and the others on Days 4–5. In all cases, retrospective analysis of the time lapse images indicated that cell number had not increased for 24–48 h (Supplementary Data Summary).

### Maternal meiotic errors identified by meiomapping

All of the polar bodies were successfully amplified by whole genome amplification (n = 102) and the DNA analysed by SNP genotyping and meiomapping to identify meiotic segregation errors^17, 18^. Excluding several meiotic structural abnormalities, 30 embryos were predicted to be normal for all maternal chromosomes and 21 embryos (41%) had abnormal meiomap patterns in the polar bodies predicting whole chromosome aneuploidy for maternal chromosomes in the corresponding embryos (Supplementary Table 1). Comparison with karyomapping of samples from the corresponding embryos, confirmed that 27/29 (93%) of the embryos predicted to be normal were euploid for all maternal chromosomes. (One trophectoderm biopsy sample failed to amplify). Of the two discordant embryos, one embryo (1(1) 2) had a maternal monosomy 22 in a single cell, and another embryo (1(2) 2) had lost 12 maternal chromosomes, presumably prior to syngamy. Of the 21 embryos predicted to have maternal aneuploidies, all were confirmed to be aneuploid. Overall, 62/62 (100%) of the predicted maternal meiotic aneuploidies (24 trisomies and 38 monosomies), ranging from 1–7 aneuploidies per embryo, were confirmed. Thus, the proportion of blastocysts and arrested embryos with confirmed maternal meiotic aneuploidies was 11/26 (42%) and 10/25 (40%), respectively, which is not significantly different.

### Chromosome profiling by karyomapping and chromosome fingerprinting

Of the 25 arrested embryos, 13 were disaggregated into single cells either completely or partially and the remainder of the embryo analysed separately. The other 12 were analysed intact. In total, 89 single cells were sampled from arrested embryos and 11 excluded cells sampled from 7 blastocysts (Supplementary Table 2). In contrast to the polar bodies, only 65/89 (73%) and 9/11 (82%) of single cells from arrested embryos and excluded cells, respectively, had detectable DNA following whole genome amplification. Furthermore, SNP genotyping of some of the products with no detectable DNA was also negative (Supplementary Data Summary).

**Figure 1:**
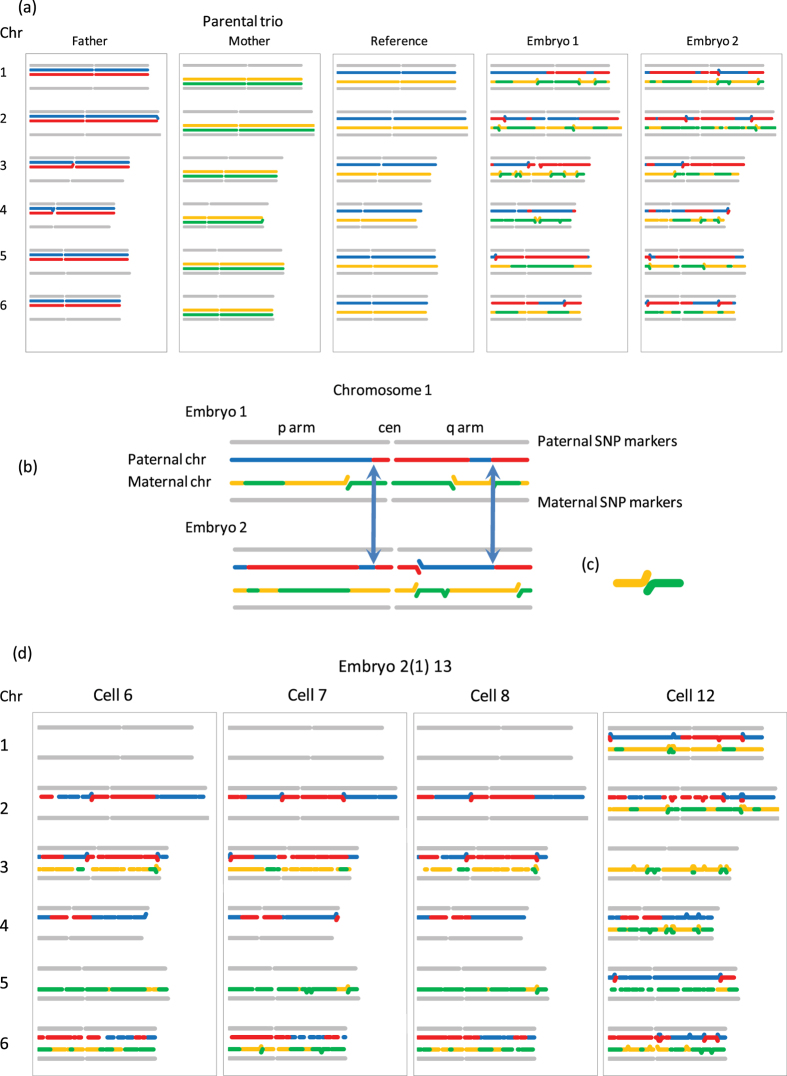
Principle of fingerprinting parental chromosomes by genome-wide karyomapping. (**a**) Following single nucleotide polymorphism (SNP) genotyping of both parents and the reference (parental trio), informative SNP loci are identified for all four parental chromosomes genome-wide (chromosomes 1–6 are illustrated here) and heterozygous informative SNPs phased using the genotype of the reference DNA which can be from an existing child, sibling embryo or, as in this example, a chorion villus sample from a previous pregnancy. The details of the algorithm have been published previously^16^. For karyomapping, the four sets of informative SNPs for the parental chromosomes are displayed as horizontal lines (left to right, pter to qter with a gap at the centromere) and colour coded: blue and red for the paternal chromosomes and orange and green for the maternal chromosomes. The reference chromosomes are defined as blue and orange. The genotype of informative SNPs in the embryo samples (Embryos 1 and 2) is then compared to the reference and displayed in the same format. Because each gamete from the parents has a unique pattern of crossovers between the parental chromosomes, as a result of recombination in the first meiotic division, each recombinant chromosome inherited by the embryo has a correspondingly unique pattern of chromosome segments or ‘fingerprint’ from the two paternal chromosomes. In these examples, all of the chromosomes are recombinant but non-recombinant patterns are also observed. (**b**) Details of the karyomap fingerprint for chromosome 1 showing different patterns of chromosomal segments on both arms for the two embryos. Note that all embryo samples compared to the reference will include any crossovers in the reference sample, which the algorithm assumes is non-recombinant, in addition to any crossovers unique to that chromosome. Two of these ‘common’ crossovers for the paternal chromosome are indicated by the arrows. (**c**) The algorithm used to display the informative SNPs present in the embryo samples uses a rolling window and where that coincides with a crossover results in this configuration indicating the presence of both sets of informative SNPs from that parent in that window. (**d**) Karyomaps and fingerprints for chromosomes 1 to 6 in four single cells disaggregated from an arrested embryo. Cells 6–8 have an identical set of 7 out of the normal 12 chromosomes, whereas cell 12 has 11 chromosomes and is only missing paternal chromosome 3. The unique pattern of parental chromosome segments for each chromosome is identical in each of the single cells where the chromosomes are present. Paternal and maternal chromosomes, blue/red and orange/green horizontal lines, respectively.

Of those single cells which did amplify, all were genotyped and karyomapped successfully, allowing each chromosome (with the exception of the Y chromosome) to be identified by the unique pattern, or ‘fingerprint’, of parental haplotypes resulting from recombination in meiosis (Fig. 1a–c). In all embryos, each of the parental chromosomes present in each cell had the same fingerprint (Fig. 1d). Also, the patterns of recombination of the maternal chromosomes were consistent with the patterns predicted by meiomapping of the polar bodies.

**Figure 2:**
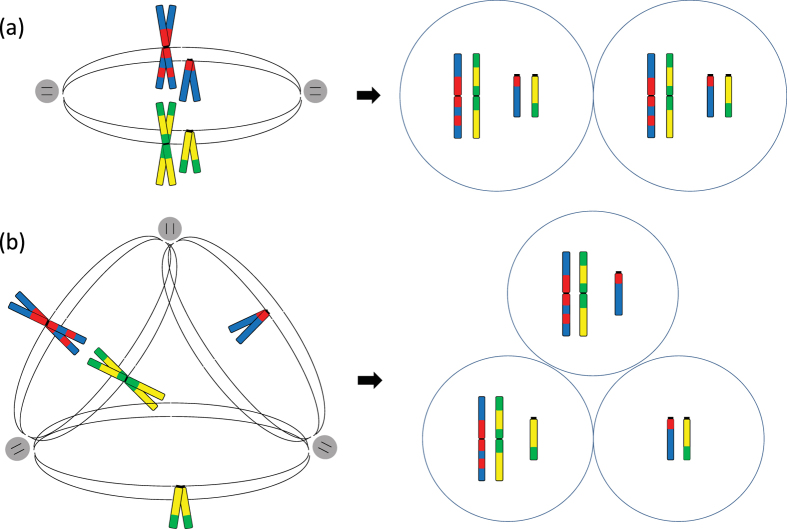
Tripolar mitosis and intercellular partitioning of the genome. Diagram comparing the segregation of one pair of metacentric and one pair of acrocentric chromosomes on (**a**) a normal bipolar spindle, and (**b**) a tripolar spindle. Paternal and maternal pairs of sister chromatids, blue/red and orange/green stripes, respectively; centrosomes, grey dots with black lines; spindle microtubules, black lines. With normal chromosome segregation on a bipolar spindle, both daughter cells have the normal two pairs of chromosomes. With tripolar mitosis and random congression of chromosomes to one of three axes, two out of three daughter cells have only 3 chromosomes and are monosomic for the acrocentric chromosome and the third cell is nullisomic for the metacentric chromosome and has a normal biparental pair for the acrocentric chromosome.

Of the 74 single cells which were karyomapped, 64 (86%) had extensive karyotype-wide genomic imbalance caused by loss of one parental chromosome (monosomy) and one or more chromosomes in which neither parental chromosome was detected (nullisomy) as would be predicted following, for example, tripolar mitosis (Figs 2 and S1a; Supplementary Table 2; Supplementary Data Summary). Thus, complete or partial genome loss was evident in 90/100 (90%) of single cells from arrested embryos and excluded cells at the blastocyst stage. The extent of this genome loss is estimated to range from 5–67%. The number of chromosomes represented in each cell was also significantly reduced (mean 27 ± 10; median 27; range 8 to 47) (Figure S1b).

In some cases, when attempting to disaggregate the cells of an arrested embryo, there was a group of small cells or cytoplasmic fragments, which could not be separated; these were analysed together as ‘fragments’. Combining the chromosomes identified in the single cells and fragments and taking into account any maternal meiotic errors, reconstituted the complete set of paternal and maternal chromosomes in all cases (Supplementary Data Summary). (There was no evidence for meiotic errors of paternal chromosomes in any of the embryos). This is strong evidence that the missing chromosomes in single cells are not a result of random mitotic errors or degradation but result from abnormal patterns of chromosome segregation and distribution among the constituent cells of the embryo. Furthermore, this is also consistent with the apparently complete chromosome profiles of intact or partial arrested embryos in which all or a majority of the constituent cells were analysed together.

### Chromosome fingerprinting identifies interrelated clones of cells

**Figure 3:**
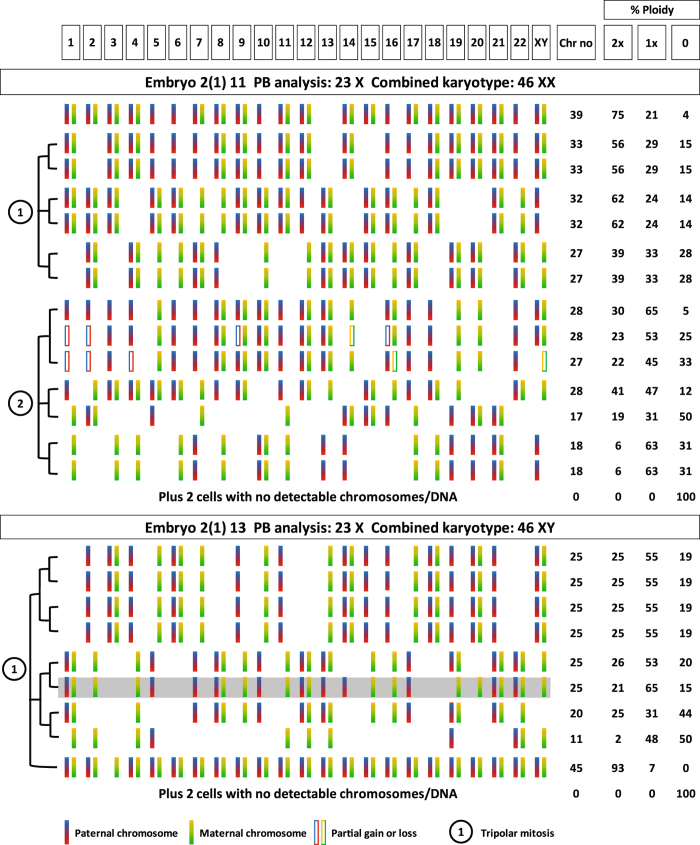
Clones of subdiploid single cells from arrested embryos following tripolar mitosis. Complete karyomap profiles of 14 and 8 single cells disaggregated from embryos 2(1) 11 and 2(1) 13, respectively, arrested at precompact morula stages on day 6 post intracytoplasmic sperm injection (ICSI). All of the cells, except two (see Results), had significantly reduced total numbers of chromosomes and, all were genomically imbalanced and had one or more nullisomies and therefore genome loss. Grouping cells with identical or complementary profiles reveals the presumed clonal lineage relationships (bracketed left). These are consistent with the time lapse observations of dual tripolar mitoses at the 2-cell stage for embryo 2(1) 11 (two groups of three) and tripolar mitosis in the first division for embryo 2(1) 13 (three groups). Note that for each of these three groups of three presumed clones, individual paternal and maternal chromosomes are only present in two out of three clones as would be predicted for normal segregation of sister chromatids on a tripolar spindle. (The profile shaded in grey is the presumed profile of one of two cells analysed together (see Results)). Also, the combined karyotype of both embryos, adding together all of the chromosomes identified in all of the embryo samples, was euploid 46, XX and 46, XY, respectively, consistent with the absence of any maternal meiotic errors detected by meiomap analysis of both polar bodies.

Close examination of the chromosome profiles identified by karyomapping in single cells from individual arrested embryos revealed many examples in which two or more cells had identical or closely related profiles providing evidence for clonal expansion following an abnormal mitotic division in which the chromosomes did not segregate equally to two daughter cells (Supplementary Data Summary). Furthermore, in two embryos, multiple clones with chromosome profiles consistent with tripolar mitosis were identified (Fig. 3). In one embryo, six sets of cells with identical, closely similar or related chromosome profiles are present consistent with two tripolar mitoses. In the other embryo, including the multiple cell fragment sample with 45 chromosomes as one component, there are three sets of cells consistent with a single tripolar mitosis. Critically in the first example, all of the parental chromosomes present are only represented in two out of the three clonal lineages providing evidence for normal disjunction following random recruitment to one of the three axes of a tripolar spindle^15^. Also, combining the chromosomes present in all of the cells completes the predicted karyotype for both sets. In the other embryo, there is one set of cells with identical chromosome profiles, one set with identical or related profiles and the remaining missing chromosomes are all present in the multiple cell fragment sample. Copy number and detailed SNP analysis revealed that this is likely to be a combination of cells with different chromosome profiles including one cell that has the shaded profile and belongs to one of the two clonal sets.

Excluded cells had a similar pattern of reduced chromosome number and closely related profiles (Figure S2; Supplementary Table 2). Furthermore, with embryos 1(1) 10 and 2(1) 9, combining the chromosomes present in all of the excluded cells almost reconstitutes the complete set identified in the corresponding trophectoderm biopsies, providing evidence that the origin of the pattern of chromosomes present was an abnormal mitotic event.

### Retrospective time lapse analysis confirms tripolar and other abnormal mitoses are associated with developmental arrest

**Figure 4:**
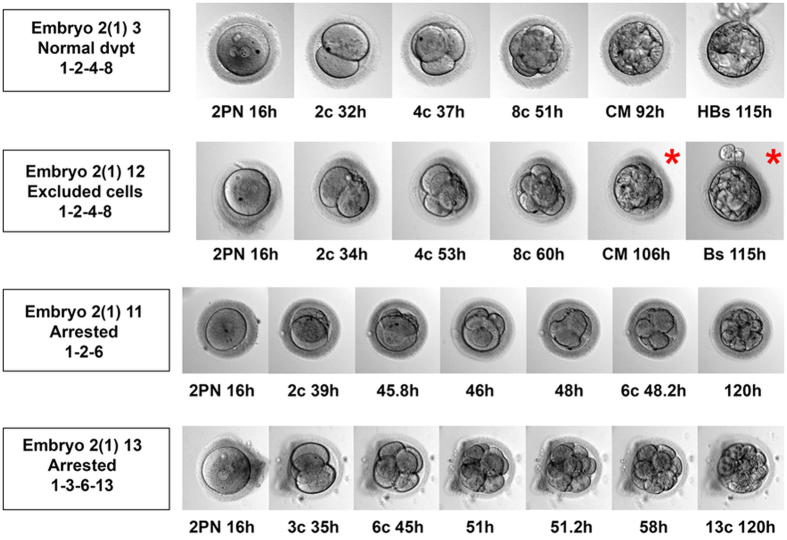
Time lapse images of normally developing and arresting embryos *in vitro*, up to day 5 (120 h) post intracytoplasmic sperm microinjection (ICSI). Time lapse imaging of embryo 2(1) 3 reveals a normal pattern of cleavage and blastocyst development, in which each of the first three cleavage divisions is completed before the next begins, resulting in distinct 1-cell, 2-cell, 4-cell and 8-cell stages (1-2-4-8). The embryo then compacts at the morula stage prior to blastocyst formation. With embryo 2(1) 12, the first three cleavage divisions progressed normally but a small number of cells were excluded from the developing blastocyst (asterisk). Embryo 2(1) 11 arrested at the precompact morula stage and both blastomeres at the 2-cell stage divided into three cells between 45.8 and 48 h. Embryo 2.13 divided from the 1-cell zygote stage into three cells and later arrested at the precompact morula stage. PN, pronuclei; 2c, 4c etc, 2-cell stage, 4-cell stage etc; CM, compact morula stage; Bs, blastocyst stage; HBs, hatching blastocyst.

Retrospective time lapse analysis showed that the normal pattern of the first three cleavage divisions of the zygote (1–2–4–8) predominated in embryos which developed to the blastocyst stage and was less frequent in arresting embryos (Fig. 4; Supplementary Data Summary). In the two embryos, with chromosome profiles consistent with tripolar mitoses, however, these were clearly evident by time lapse. In embryo 2(1) 11, which had two sets of three clones, tripolar mitosis was observed for both blastomeres at the 2-cell stage in the second division. With the other embryo 2(1) 13, the zygote divided directly into three cells. Also, in some embryos with excluded cells at the blastocyst stage, it was possible to identify abnormal mitoses in the later cleavage divisions and to follow the cells at later stages. These cells failed to be incorporated when the embryo compacted at the morula stage and remained on the outer surface of the embryo at the blastocyst stage.

## Discussion

Combining genome-wide SNP genotyping and meiomapping of polar bodies to identify maternal meiotic errors and karyomapping for chromosome fingerprinting has enabled the distribution of parental chromosomes in the constituent cells of the human preimplantation embryo to be examined. This has allowed the effects of meiotic versus mitotic aneuploidies on developmental arrest to be studied and clonal cell lineages with identical or related abnormal chromosome profiles to be identified in arrested embryos. Previous studies examining chromosome copy number in single cells from disaggregated embryos also identified possible clonal relationships between cells but were limited to a subset of chromosomes analysed by fluorescence *in situ* hybridisation (FISH)^19^ or array comparative genomic hybridisation (array CGH) analysis of all chromosomes in selected normally developing cleavage stage embryos^20, 21^. Copy number analysis by array CGH or, next generation sequencing (NGS) based methods, are designed to detect genomic imbalance in small numbers of chromosomes. Copy number is normalised to a baseline set by the majority of chromosomes and assumed to be two copies. Thus, extensive imbalance and nullisomy can result in chaotic patterns which are difficult to interpret and may be considered technical failures. Whilst karyomapping *per se* cannot detect multiple copies of the same chromosome, it has the advantage that it can detect even small numbers of chromosomes accurately from the recombinant fingerprint. In this study, karyomapping was able to detect as few as 8 chromosomes in a single cell (Supplementary Data Summary).

Chromosome mosaicism was first observed in human cleavage stage embryos using multicolour fluorescence *in situ* hybridisation (FISH) to interphase nuclei, with probes for limited numbers of chromosomes^4, 5^. More recently, these observations have been extended by array CGH and copy number analysis for all chromosomes in disaggregated normally developing embryos at the 8-cell stage^20, 21^. What is remarkable about the chromosome mosaicism identified here is (1) the characteristic pattern of karyotype-wide aneuploidies which frequently includes a combination of biparental disomy, paternal and maternal monosomies and nullisomies, (2) identical or closely similar chromosome profiles are present in two or more cells from some embryos providing evidence of their clonal origin, (3) combining the chromosomes present in each cell from disaggregated embryos completes the expected karyotype, and finally (4) the pattern of chromosomes in different cells or clones is often interrelated or complementary. Specifically, we have identified three examples of almost complete sets of clones and single cells consistent with tripolar mitosis, one in the first cleavage division in one embryo and two in the second division of another embryo (Fig. 3).

Taken together these features provide strong evidence that, despite the apparently chaotic chromosome profiles in each cell, chromosome segregation, i.e. the bipolar segregation of sister chromatids following DNA replication, occurs normally throughout cleavage prior to developmental arrest. However, one or more abnormal mitotic events, particularly tripolar mitoses as identified here, causes the diploid chromosome set to be partitioned between cells resulting in karyotype-wide genetic imbalance, nullisomies and therefore partial genome loss in the affected lineages. If this were to occur in the first division (or in both cells at the second division), all of the cells of the embryo will be affected, whereas, if it were to occur in later divisions, only a part of the embryo will be affected. This may explain why some embryos arrest whilst others fail to incorporate some cells at the blastocyst stage. For example, in one embryo 2(1) 12, a late tripolar mitosis was observed by time lapse imaging and correlated with the position of several excluded cells two of which had identical chromosome profiles including multiple monosomies and two nullisomies (Fig. 4; Supplementary Data Summary).

Time lapse imaging and retrospective analysis of the development of a large series of embryos has shown that the incidence of abnormal mitoses, including tripolar mitosis, in the first three cleavage divisions was 10%, 9% and 4%, respectively, and a further 4% had multiple abnormal mitoses, which overall is 26% per embryo^14^ confirming an earlier study on a smaller series of embryos which identified abnormal mitoses in 24% of embryos^13^. Furthermore, abnormal mitoses were correlated with developmental arrest prior to blastocyst formation, increased chromosome aneuploidy and poor clinical outcomes following transfer. Retrospective time lapse analysis of the first three cleavage divisions in the embryos in this study showed a general correlation of the normal pattern (1-2-4-8) with development to the blastocyst stage and conversely, developmental arrest if the zygote divided into 3 or more cells in the first division (Supplementary Data Summary). However, a limitation of time lapse imaging by light microscopy is that the cellular events cannot be directly correlated with nuclear division and chromosome segregation. That will require live cell, laser confocal fluorescence microscopy and time lapse imaging with labelling of chromatin in embryos donated for research^22^.

Most of the arrested embryos examined in this study, arrested on days 4 to 5 post insemination at cleavage stages before compaction and the morula-blastocyst transition. The median estimated cell number in arrested embryos was 13, which together with the observations of tripolar mitosis and number of cells in recognised clones, indicates that these embryos had completed the first three cleavage divisions before developmental arrest. For example, embryo 2(1) 13 underwent tripolar mitosis in the first division and four cells with identical chromosome profiles provide evidence for two further divisions before arrest (Fig. 3). Prior to zygotic genome activation (ZGA), the embryo is dependent on maternal inherited proteins and RNAs. With the human embryo, degradation of maternal transcripts and ZGA is thought to occur between the 4- and 8-cell stages since inhibition of transcription blocks development beyond these stages^23^. More recently, detailed RNA sequencing studies of embryos and single cells have broadly confirmed the timing of this transition and identified panels of lineage specific genes^24, 25^. It seems likely therefore that development is arrested prior to compaction as a consequence of the extensive genomic imbalance and partial genome loss at the cellular level and disruption of the normal pattern of ZGA affecting a broad range of cellular processes including those known to be highly coordinated during compaction, such as cell-cell adhesion and the establishment of apicobasal polarity^26^. This has important consequences for studies of gene expression at cleavage stages in which unrecognised genomic imbalance and nullisomies may affect normal expression patterns^27^ With the development of methods for parallel DNA and RNA sequencing from single cells^28^, it may be possible to study the relationship between the partitioned genome and the transcriptome in affected cells.

The primary event associated with the developmental arrest at late cleavage stages observed in the embryos in this study was one or more tripolar or other abnormal mitoses, which secondarily resulted in the dispersal of the normal diploid set of chromosomes, effectively partitioning the genome between cells. In the mouse, knockout of a broad spectrum of cell cycle related genes causes an increased incidence of aneuploidies, chromosome breakage and other defects at preimplantation stages^29^. However, unless mitosis is blocked, embryo development generally proceeds to the blastocyst stage and the mutation(s) only become lethal in the peri-implantation period. Recently, complex aneuploidies (multiple gains and losses) of mitotic origin identified in a large series of single blastomeres and trophectoderm cell samples biopsied from cleavage and blastocyst stage embryos, by SNP genotyping and haplotype analysis similar to karyomappping, were shown to be associated with a common maternal variant in a region of chromosome 4 which includes the gene polo-like kinase 4, *PLK4* ^30^. *PLK4* plays a critical role in centrosome duplication and the centrosome cycle^31^. Furthermore, these complex mitotic aneuploidies were much less frequent in trophectoderm samples providing evidence that they were selected against before the blastocyst stage^10^. Interestingly, two of the three patients with examples of tripolar mitosis were homozygous for the high risk SNP allele (AA; rs2305957) (Table 1).

Chromosome aneuploidy of meiotic and mitotic origin is a major cause of pregnancy loss and abnormal pregnancies, and is only rarely compatible with development to live birth^8^. Following embryo transfer at the blastocyst stage, aneuploidy is also associated with IVF failure demonstrating that genomic imbalance of one or more chromosomes is sufficient to prevent implantation and/or block development at peri-implantation stages^9^. It now appears that in addition to isolated anaphase lag or non-disjunction events, karyotype-wide aneuploidies including monsomies and nullisomies arising from tripolar and other abnormal mitoses in early cleavage may cause developmental arrest and block the morula-blastocyst transition in a significant proportion of human preimplantation embryos *in vitro*. Thus, all adverse outcomes following human conception, at least *in vitro*, may have an underlying genetic basis.

## Methods

### Source of human embryos

Three couples presented with either an inherited genetic disorder and/or previous failed IVF treatment and requested either preimplantation genetic diagnosis (PGD) of a single gene defect by SNP genotyping and karyomapping or preimplantation genetic screening (PGS) for aneuploidy by array CGH or NGS-based copy number analysis.

### Informed consent

All of the data used in this study was generated in the course of clinical treatment involving the genetic testing of human embryos for a single gene defect and/or aneuploidy screening in a private IVF clinic licensed by the Human Fertilisation and Embryology Authority in the UK and in accordance with all relevant regulations and legislation. Follow up analysis of genetically tested embryos is established best practice for quality control purposes. All patients and their partners were counselled both by the clinician managing their treatment and a genetic counsellor and provided written informed consent for the genetic testing and follow up analysis.

### Egg collection and embryo culture

A standard short cycle gonadotrophin releasing hormone (GnRH) antagonist regimen was used for controlled ovarian hyperstimulation and transvaginal ultrasound-guided eggs collection performed approximately 36 h after administration of human chorionic gondaotrophin (hCG). Following removal of the cumulus cells, mature metaphase II eggs underwent biopsy of the first polar body (PB1) and were inseminated by intracytoplasmic sperm microinjection (ICSI) using micromanipulation, as previously described^17^. Injected eggs were cultured individually in microwell dishes (GeneaBiomedx, Australia) overlaid with 80 µl medium (Continuous Single Culture Medium (CSCM); Irvine, USA) under oil (Ovoil; Vitrolife, Sweden) at 37 °C in a time lapse incubator (Geri; GeneaBiomedx, Australia) in an atmosphere of 6% CO_2_ and 5% O_2_. Fertilisation was checked the following day (16–18 h later), by examining the time lapse video recording for extrusion of the second polar body (PB2) and the presence of two pronuclei, and the PB2 biopsied. Fertilised embryos were then returned to the time lapse incubator and cultured continuously to day 5 or 6 post insemination and morphological development monitored by time lapse recording. Abnormally fertilised embryos (with abnormal numbers of pronuclei) were excluded from this study.

### Trophectoderm biopsy and vitrification

Embryos that developed to the expanded blastocyst stage on days 5–7 post insemination had 3–6 trophectoderm cells biopsied by micromanipulation for clinical genetic testing, as described previously^32^. Any excluded cells remaining on the surface of the blastocyst were also removed by micromanipulation and collected separately. The biopsied blastocysts were then vitrified for later clinical use.

### Arrested embryo disaggregation

Embryos which had shown no evidence of cell division for the preceding 24 h as assessed by time lapse analysis and had not developed to the blastocyst stage by day 6 post insemination were considered arrested. The zona pellucida of each arrested embryo was first thinned by brief exposure to acidified Tyrode’s solution (Origio,USA) and removed by gentle pipetting. The arrested embryos were then prepared for genetic analysis either whole or following disaggregation for single cell analysis (see Supplementary data for details). For disaggregation, embryos were incubated in 10 µl drops of Ca^++^ Mg^++^ free medium (Embryo biopsy medium; Irvine, USA) and vigorously pipetted.

### Sample preparation, DNA extraction and Whole Genome Amplification (WGA)

All embryo samples including biopsies, whole embryos and single cells disaggregated from arrested embryos were washed through several drops of phosphate buffered saline (PBS) (Gibco; Life technologies, USA) supplemented with 0.1% polyvinyl alcohol (Sigma-Aldrich, USA) and placed into PCR tubes in 10 µl of PBS. DNA extraction from buccal cell swabs from both parents and close relatives and lysis of embryo samples was as described previously^17, 18^. Whole genome amplification (WGA) of embryo samples was by a PCR library based method according to manufacturer’s instructions (SurePlex, Illumina, San Diego, USA).

### SNP genotyping

400 ng of genomic DNA or 8 µl of WGA products from the single cell and embryo samples were processed on a beadarray for genome-wide genotyping of approximately 300 K single nucleotide polymorphism (SNP) markers (Human Karyomapping-12; Illumina, San Diego, USA).

### Meiomapping of polar bodies

Following whole genome amplification and SNP genotyping of the first and second polar bodies (PB1 and PB2), meiomap analysis was performed using a dedicated VBA macro in Microsoft Excel as previously described^17, 18^. Abnormal patterns of chromosome segregation in the two meiotic divisions were based on analysis of the maternal haplotype patterns in PB1 and PB2. As meiomap analysis of the polar bodies alone cannot distinguish the presence of 3 or 4 chromatids in PB1, the segregation pattern was identified by reference to the karyomap of the corresponding embryo. Comparison of the patterns of recombination in polar bodies and embryo samples was analysed by karyomapping and the predicted pattern was always concordant with the karyomap of the maternal chromosome(s) in the corresponding embryo.

### Karyomapping of embryo samples

Karyomaps of polar bodies and embryo samples were processed from SNP genotype data using a dedicated VBA macro as previously described^16^. Karyomaps for all 22 autosomes and the X chromosome were then displayed using a second macro to analyse the pattern of recombination and fingerprint all parental chromosomes (with the exception of the Y chromosome) (Fig. 1). This was based on the proportion of informative SNPs for the four parental haplotypes in successive groups of 35 SNPs across each chromosome.

### Time-lapse analysis

The time lapse videos of all embryos were annotated for the timing and type of cell division using the manufacturer’s software (Geri Connect; GENEA, Australia). Still images were then captured at several time points in the development of each embryo: (1) the formation of pronuclei, (2) first cleavage division, (3) second cleavage division, (4) third cleavage division, (5) cell compaction, (6) cavitation, (7) expanded blastocyst, and (8) final time-lapse image prior to biopsy, disaggregation or tubing. Additional images were recorded of any tripolar mitotic events, demonstrating 3 cleavage furrows. The division pattern was recorded for each embryo in terms of how many cells were present after each division to indicate normal (i.e. 1-2-4-8 cells) or abnormal cleavage patterns (for example. 1-3-6-12 cells). The final cell number for each arrested embryo was also estimated using the Z stack facility to focus through the embryo.

### Data availability

All data generated and analysed during this study are included in this published article (see Supplementary Data Summary and Tables).

## Electronic supplementary material

Supplementary Information 

Supplementary Data Summary
