# Supplementary material for: Tripolar mitosis and partitioning of the genome arrests human preimplantation development in vitro
Source: Sci Rep. 2017 Aug 29;7:9744. doi: 10.1038/s41598-017-09693-1 (PMC5575028; doi:10.1038/s41598-017-09693-1)
Supplement: Supplementary file 1 — Supplementary Information [file 41598_2017_9693_MOESM1_ESM.pdf]

Tripolar mitosis and partitioning of the genome arrests human preimplantation development *in vitro*

Christian S. Ottolini[1,2,3], John Kitchen[4], Leoni Xanthopoulou[5], Tony Gordon[5], Michael C Summers[1,2] and Alan H Handyside[1,2*]

[1]The Bridge Centre, One St Thomas Street, London SE1 9RY, UK

[2]School of Biosciences, University of Kent, Canterbury CT2 7NJ, UK

[3]London Women's Clinic, 113-115 Harley Street, Marylebone, London W1G 6AP, UK

[4]Genesis Genetics, 705 South Main Street, Plymouth, MI 48170, USA

[5]Genesis Genetics, London Bioscience and Innovation Centre, 2 Royal College Street, London NW1 0NH, UK

*Author for correspondence:

Prof Alan H Handyside

The Bridge Centre

One St Thomas Street

London SE1 9RY

UK

T: +44 7714 236623

Email: ahandyside@thebridgecentre.co.uk

Keywords: Meiomapping/Karyomapping/aneuploidy/chromosome mosaicism/tripolar mitosis

Figure S1: Distribution of chromosomes identified in single cells disaggregated from arrested embryos or excluded from embryos at the blastocyst stage

(a) The incidence of biparental disomy, paternal and maternal monosomy and nullisomy in single cells for each of the autosomes and the sex chromosomes (n=73).(b) The total number of parental chromosomes identified per single cell. The actual copy number of chromosomes may be greater if more than one copy is present in a particular cell. Also, chromosomes with structural abnormalities (partial gain or loss) are included.

(a)

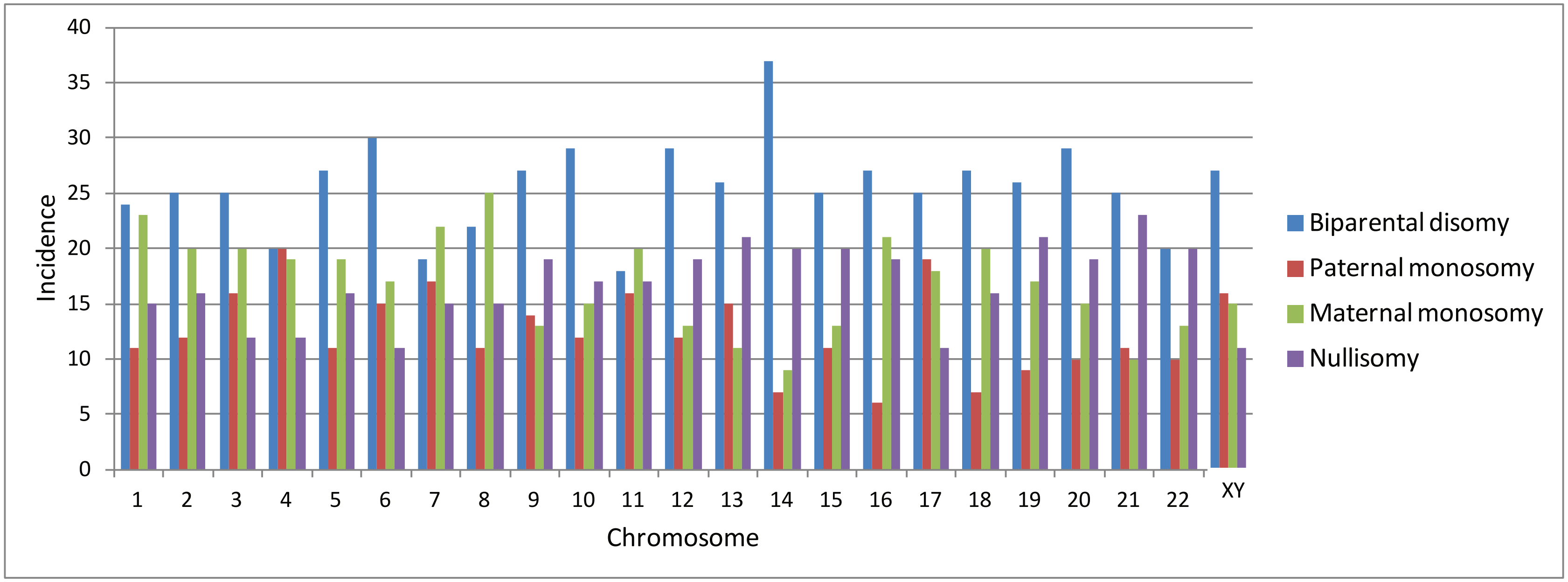

(b)

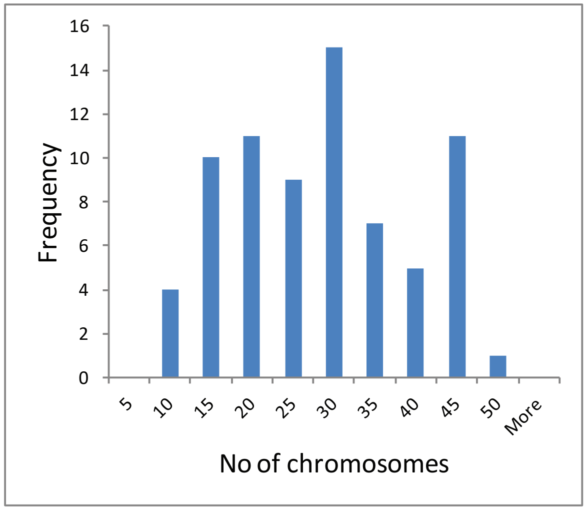

Figure S2: Karyomap profiles of single cells excluded from the embryo at the blastocyst stage
Complete karyomap profiles of nine single cells excluded from four poor quality blastocyst stage embryos on day 6 post ICSI. As with the cells from arrested embryos, these excluded cells all had significantly reduced total numbers of chromosomes and, all were genomically imbalanced and had one or more nullisomies and genome loss. Within the groups of two or three excluded cells from all four embryos, there are similarities or complementarity in the profiles indicating possible late multipolar divisions as their origin.

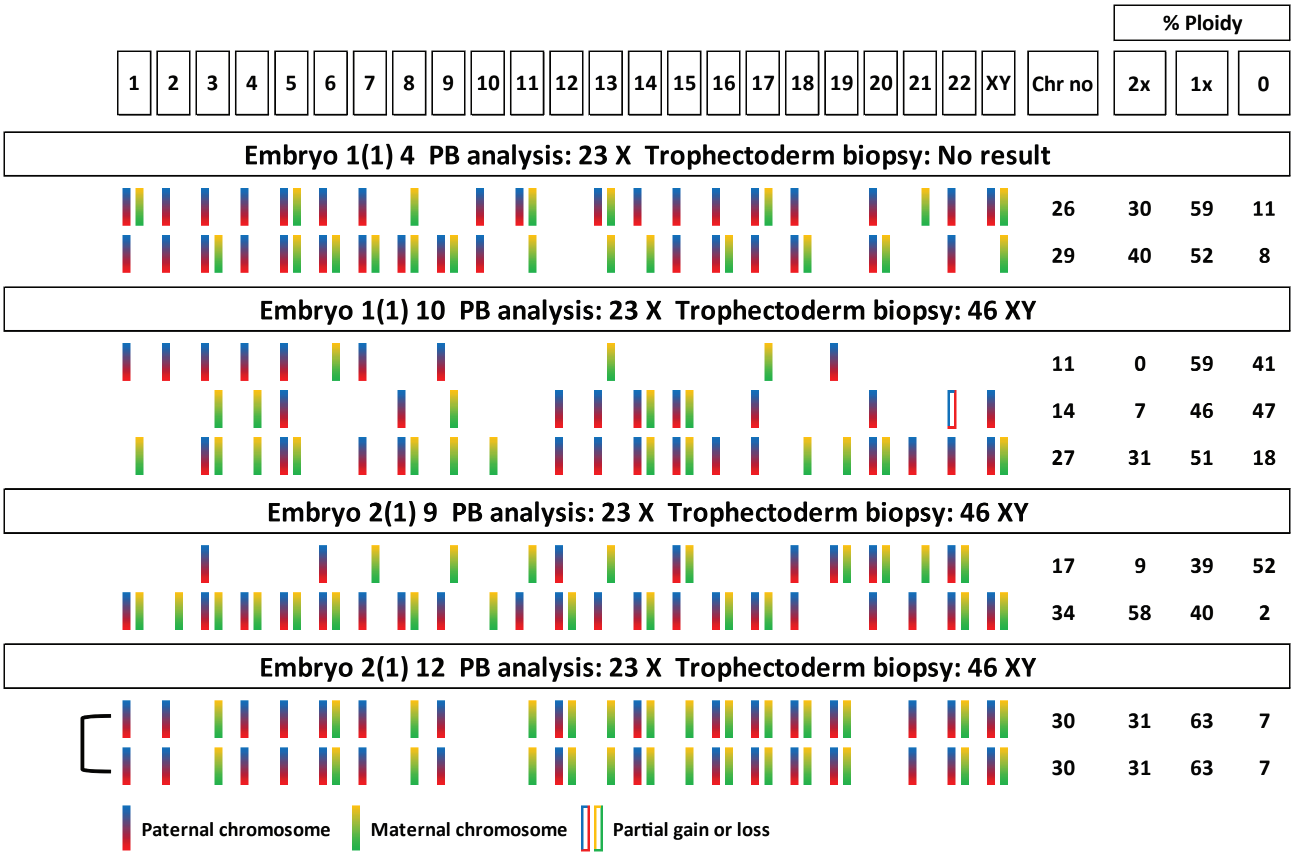

Supplementary Table 1: Comparison of maternal chromosome complement predicted by meiomapping of polar bodies and karyomapping of blastocyst stage and arrested embryos (excluding analysis of meiotic structural abnormalities)

Patient (Cycle)	Embryo #	Day post ICSI	Meiomap	Karyomap Paternal	Karyomap Maternal	Type of embryo sample
**Blastocysts n=26**						
**1 (1)**	**2**	7	Euploid	Euploid	-19	Emb
	**4**	5	Euploid	No result		Biopsy
	**5**	5	Euploid	Euploid		Biopsy
	**7**	5	+16	Euploid	+16	Biopsy
	**8**	5	+14 -22	Euploid	+14 -22	Biopsy
	**10**	5	Euploid	Euploid		Biopsy
**1 (2)**	**1**	6	Euploid	Euploid		Biopsy
	**4**	5	Euploid	Euploid		Biopsy
	**8**	6	-8, 16	Euploid	-8, 16	Biopsy
	**11**	5	Euploid	Euploid		Biopsy
	**14**	5	Euploid	Euploid		Biopsy
	**15**	6	-16	Euploid	-16	Emb x2
	**16**	5	Euploid	Euploid		Biopsy
**2 (1)**	**2**	5	Euploid	Euploid		Biopsy
	**3**	5	Euploid	Euploid		Biopsy
	**4**	5	Euploid	Euploid		Biopsy
	**9**	5	Euploid	Euploid		Biopsy
	**10**	5	Euploid	Euploid		Biopsy
	**12**	5	Euploid	Euploid		Biopsy
**3 (1)**	**1**	5	+2, 7, 10, 14<br>-12, 21	Euploid	+2, 7, 10, 14<br>-12, 21	Emb
	**4**	5	-3, 22	Euploid	-3, 22	Biopsy
	**6**	5	-9, 15	Euploid	-9, 15	Biopsy
	**9**	6	+16	Euploid	+16	Biopsy
	**17**	5	+13	Euploid	+13	Biopsy
	**18**	5	-10, 11, 18	Euploid	-10, 11, 18	Biopsy
	**19**	5	+13, 18 -2, 8	Euploid	+13, 18 -2, 8	Biopsy

Supplementary Table 1 (continued): Comparison of maternal chromosome complement predicted by meiomapping of polar bodies and karyomapping of blastocyst stage and arrested embryos (excluding analysis of meiotic structural abnormalities)

Patient (Cycle)	Embryo #	Day post ICSI	Meiomap	Karyomap		Type of embryo sample
				Paternal	Maternal	
**Arrested embryos n=25**						
**1 (1)**	**3**	6	Euploid	Euploid	Euploid	Single cells
	**6**	7	-2	Euploid	-2	Whole
	**9**	6	-1, 5, 8, 9, 13, 16, +X	Euploid	-1, 5, 8, 9, 13, 16, +X	Single cells
	**11**	7	Euploid	Euploid		Whole
	**12**	6	+4	Euploid	+4	Single cells
**1 (2)**	**2**	6	Euploid	Euploid	-1, 2, 4, 6-8, 10, 15, 16, 18-20	Single cells and Partial
	**3**	6	Euploid	Euploid		Partial
	**5**	6	Euploid	Euploid		Partial
	**6**	6	Euploid	Euploid		Whole
	**7**	6	Euploid	Euploid		Whole
	**9**	6	Euploid	Euploid		Partial
	**10**	6	Euploid	Euploid		Partial
	**13**	6	Euploid	Euploid		Partial
**2 (1)**	**6**	6	-3, 10 +16	Euploid	-3, 10 +16	Whole
	**7**	6	Euploid	Euploid		Whole
	**8**	6	Euploid	Euploid		Whole
	**11**	5	Euploid	Euploid		Single cells
	**13**	5	Euploid	Euploid		Single cells
**3 (1)**	**5**	6	-3, 8, 20 +22	Euploid	-3, 8, 20 +22	Single cells
	**8**	6	+16, 19	Euploid	+16, 19	Whole
	**10**	6	-1, 16, 20	Euploid	-1, 16, 20	Whole
	**12**	6	-4 +11, 15, 21, 22		-4 +11, 15, 21, 22	Single cells
	**13**	6	-1, 21 +22	Euploid	-1, 21 +22	Whole
	**15**	6	Euploid	Euploid		Whole
	**16**	6	-2, 11, 16, 19 +18, 22	Euploid	-2, 11, 16, 19 +18, 22	Single cells

Supplementary Table 2: Amplification efficiency and genome loss in single cells from arrested embryos and excluded at the blastocyst stage

Patient (Cycle)	Embryo #	Day	Single cells sampled	No amp/DNA	Amp	No. with ≥1 nullisomies	No. with genome loss
**Disaggregated arrested embryos**							
**1 (1)**	**3**	6	7	4	3	3	7
	**9**	6	12	3	9	9	12
	**12**	6	9	5	4	4	9
**1 (2)**	**2**	6	3	0	3	3	3
	**3**	6	3	2	1	1	3
	**5**	6	3	1	2	2	3
	**9**	6	3	2	1	1	3
	**10**	6	3	0	3	3	3
	**13**	6	3	2	1	0	2
**2 (1)**	**11**	5	16	2	14	14	16
	**13**	5	9	2	7	7	9
**3 (1)**	**5**	6	4	0	4	0	0
	**12**	6	7	1	6	3	4
	**16**	6	6	0	6	5	5
**Total (%)**	**14**		**88**	**24 (27)**	**64**	**55 (85)**	**79 (90)**
**Excluded single cells at the blastocyst stage**							
**1 (1)**	**4**	5	3	1	2	2	3
	**8**	5	1	1	0	-	1
	**10**	5	4	1	3	3	4
**1 (2)**	**15**	6	1	0	1	1	1
**2 (1)**	**4**	5	1	1	0	-	1
	**9**	5	1	0	1	1	1
	**12**	5	2	0	2	2	2
**Total (%)**	**7**		**13**	**4 (31)**	**9**	**9 (100)**	**13 (100)**
**Combined total (%)**	**21**		**101**	**28 (28)**	**74**	**64 (86)**	**92 (91)**
